# Public Awareness and Knowledge of Pap Smear as a Screening Test for Cervical Cancer among Saudi Population in Riyadh City

**DOI:** 10.7759/cureus.984

**Published:** 2017-01-17

**Authors:** Hassan Al Khudairi, Ahmed Abu-Zaid, Osama Alomar, Hany Salem

**Affiliations:** 1 Obstetrics & Gynecology, King Saud Medical City (Saudi Arabia); 2 College of Medicine, Alfaisal University; 3 Obstetrics & Gynecology, King Faisal Specialist Hospital and Research Center Riyadh (Saudi Arabia)

**Keywords:** cervical cancer, pap smear, screening, prevention, awareness, riyadh, saudi arabia

## Abstract

Aims: To explore the public awareness, knowledge, and attitudes of Saudi women towards Pap smear as a screening test for cervical cancer.

Methods: A descriptive cross-sectional study took place in four major secondary and tertiary healthcare hospitals located in the capital city Riyadh between January 2016 and June 2016. A self-administered, coded, close-ended survey was randomly distributed to 1000 non-single women attending the obstetrics/gynecology outpatient clinics or inpatient wards.

Results: Five hundred and seven women participated in the survey (overall response rate: 50.7%). The vast majority of respondents aged between 20-40 years (88%) and were married (94.1%), Saudi citizens (96.5%), university educated (45.6%) and housewives (64.5%). A total of 234 women (46.2%) did not hear whatsoever about Pap smear previously. Only 273 women (53.9%) heard about it, mostly during their hospital visits for obstetric/gynecologic purposes (57.1%). A sum of 381 women (75.2%) did not do a single Pap smear previously. A sum of 383 women (75.5%) reported that their physicians never advised them to do Pap smear. Regarding knowledge of Pap smear, 415 women (82%) did not know when to start doing Pap smear, 471 women (92.9%) did not know how frequently they should do Pap smear and 476 women (93.9%) did not know when to stop doing Pap smear. Moreover, 456 women (89.9%) did not know the difference between Pap smear and high vaginal swap. A total of 429 women (84.6%) never requested their physician to do Pap smear. Almost all women (95.3%) expressed an interest in knowing more information about the Pap smear screening test.

Conclusion: The awareness and knowledge of Pap smear as a screening test for cervical cancer among Saudi population living in Riyadh is unsatisfactory. There is an urgent necessity to educate and foster awareness concerning cervical cancer and its screening through Pap smear.

## Introduction

In Saudi Arabia, cervical cancer is ranked the ninth most common cancer in Saudi females [1]. Moreover, it comprises approximately 2.6% of all newly diagnosed cancers in Saudi females [1]. Human papillomavirus (HPV) serotypes 16 and 18 have been established as the most frequent etiologies implicated in the development of cervical cancer [2-9].

In developed countries that employ intensive cervical cancer screening programs (that is, Pap smear), the incidence and mortality rates of cervical cancer have been reduced by roughly 70-80% [10-11]. This cytological office-based screening test can identify pre-invasive as well as invasive disease lesions at early stages of the clinical course, during which these lesions can be prevented and managed before progression. For that reason, Pap smear is universally recommended for all sexually active women [12].

In Saudi Arabia, despite the well-acknowledged benefits of Pap smear and its availability in various healthcare facilities, an intensive screening program for cervical cancer is not well established [13]. Moreover, despite the availability of HPV vaccines in the market and healthcare facilities, no formal nationwide campaigns to vaccinate females are commenced [14]. As a result, it has been observed that the number of cases of cervical cancer has been increasing over the past two decades [13].

In Saudi Arabia, there are limited studies that have endeavored to explore the awareness, knowledge, and attitudes of women towards Pap smear [13,15]. Only one study has been conducted in the capital city Riyadh and involved only university students [14]. To the best of our knowledge, no study has been conducted in the capital city Riyadh surveying public women attending healthcare facilities. It is very important to explore these domains (that is, level of awareness, knowledge, and attitude) so that positive perceptions can be reinforced and gaps can be filled in. Afterward, effective national strategies can be put into practice to increase the awareness of routine Pap smear to all sexually active women in the country.

The aim of this study was to assess the level of public awareness, knowledge and attitudes towards Pap smear as a screening test for cervical cancer among Saudi women, attending major healthcare facilities in the capital city Riyadh. Informed consent was obtained from the patient for this study.

## Materials and methods

The descriptive cross-sectional study took place in four major secondary and tertiary healthcare facilities located in the capital city Riyadh between January 2016 and June 2016. The healthcare facilities comprised: King Faisal Specialist Hospital and Research Centre, King Fahad Medical City, King Saud Medical City and Security Forces Hospital. The study was approved by the Institutional Review Board (IRB) and Research Advisory Council (RAC) at King Faisal Specialist Hospital & Research Centre, Riyadh, Saudi Arabia.

A self-administered, coded, close-ended survey was randomly distributed to 1000 non-single women attending the obstetric/gynecology outpatient clinics or inpatient wards. Exclusion criteria included incomplete surveys. The survey was created by two physicians (authors) based on a literature review and directed by the study aims. It was then peer-reviewed by another two physicians (authors) and two non-physicians (not authors) to double-check its proper content. Survey questions were originally prepared in the English language and translated into the Arabic language by two linguistic experts (not authors). It was then pre-tested on a group of women (n=15) to examine its validity and ensure proper interpretation of questions. Results of the piloted questions appeared to be satisfactory and valid. Whenever needed, explanations to patients were provided by Arabic-speaking social workers, health educators, and nurses.

 The survey was composed of four sections. The first section examined socio-demographic data, namely: age, marital status, nationality, the level of education and profession. The second section examined awareness of Pap smear as a screening test for cervical cancer. Awareness questions comprised: do you know the importance of Pap smear, have you heard about it (if yes, how did you hear about it), have you done it previously and does your physician advise you to do it. The third section examined knowledge of Pap smear as a screening test for cervical cancer. The knowledge questions comprised: do you know when to start doing Pap smear (if yes, indicate when), do you know how frequently you should do it (if yes, indicate how frequently), do you know when you should stop doing it (if yes, indicate when) and do you know the difference between Pap smear and high vaginal swap. The fourth section examined some perceived attitudes towards Pap smear as a screening test for cervical cancer. The attitude questions comprised: do you advise your family/relatives/friends to do Pap smear, have you ever requested your physician to do Pap smear and are you interested in knowing more information about Pap smear.

 All categorical data were presented as numbers and percentages.

## Results

Five hundred and fifty women participated in the survey (n=550/1000). Forty-three surveys were excluded due to the incompletion. The overall response rate was 50.7% (n=507/1000).

The vast majority of respondents aged between 20-40 years (88%) and were married (94.1%), Saudi citizens (96.5%), university educated (45.6%) and housewives (64.5%). Table 1 shows the socio-demographical data of all respondents.

**Table 1:** Socio-demographical data of all respondents (n=507)

Age	
<20	17 (3.4%)
20-29	282 (55.6%)
30-39	161 (31.8%)
40-49	35 (6.9%)
50 or more	12 (2.4%)
Marital Status	
Married	477 (94.1%)
Divorced	17 (3.4%)
Widow	13 (2.6%)
Nationality	
Saudi	489 (96.4%)
Non-Saudi	18 (3.6%)
Level of education	
Illiterate (non-educated)	23 (4.5%)
Up to intermediate school	97 (19.1%)
High school	156 (30.8%)
University	231 (45.6%)
Profession	
Student	9 (1.8%)
Housewife	327 (64.5%)
Employee	171 (33.7%)

Table 2 shows the awareness of all respondents about Pap smear as a screening test for cervical cancer. A total of 234 women (46.2%) did not hear whatsoever, about Pap smear previousl, only 273 women (53.9%) heard about it, mostly during their hospital visits for obstetric/gynecologic purposes (57.1%). The importance of Pap smear was not known to 312 women (61.5%). A total of 381 women (75.2%) did not do a single Pap smear previously. A sum of 383 women (75.5%) reported that their physicians never advised them to do Pap smear.

**Table 2:** Awareness of all respondents about Pap smear as a screening test for cervical cancer

Do you know the importance of Pap smear (n=507)	
Yes	195 (38.5%)
No	312 (61.5%)
Did you hear about Pap smear (n=507)	
Yes	273 (53.8%)
No	234 (46.2%)
If [yes], how did you hear about Pap Smear (n=273)	
Through healthcare facility and staff	42 (15.4%)
During hospital visit for obstetric/gynecologic reasons	156 (57.1%)
Through media	27 (9.9%)
Through posters, leaflets and pamphlets	23 (8.4%)
Through friends	25 (9.2%)
Have you done Pap smear before (n=507)	
Yes	126 (24.9%)
No	381 (75.1%)
Does your physician advise you to do Pap smear (n=507)	
Sometimes	79 (15.6%)
Always	45 (8.9%)
Never	383 (75.5%)

Table 3 shows the knowledge of all respondents about Pap smear as a screening test for cervical cancer. A total of 415 women (82%) did not know when to start doing Pap smear. Among those who subjectively assumed knowledge about when to start doing Pap smear (n=92), only 39 women (42.4%) answered correctly as after marriage. A total of 471 women (92.9%) did not know how frequently they should do Pap smear. Among those who subjectively assumed knowledge about the frequency of Pap smear (n= 6), only three women (8.3%) answered correctly as every three years. A total of 476 women (93.9%) did not know about when to stop doing the Pap smear screening. Among those who subjectively assumed knowledge about when to stop doing Pap smear screening (n=31), only nine women (29%) answered correctly as at the age of 65 years. Moreover, 456 women (89.9%) did not know the difference between Pap smear and high vaginal swap.

**Table 3:** Knowledge of all respondents about Pap smear as a screening test for cervical cancer

Do you know when to start doing Pap smear (n=507)	
Yes	92 (18.1%)
No	415 (81.9%)
If [yes], please indicate when (n=92)	
After marriage	39 (42.4%)
At 30 years of age	28 (30.4%)
At 40 years of age	25 (27.2%)
Do you know how frequently you should do Pap smear (n=507)	
Yes	36 (7.1%)
No	471 (92.9%)
If [yes], please indicate how frequently (n=92)	
Every six months	15 (41.7%)
Every one year	18 (50%)
Every three years	3 (8.3%)
Do you know when to stop doing Pap smear (n=507)	
Yes	31 (6.1%)
No	476 (93.9%)
If [yes], please indicate when (n=31)	
At 50 years of age	9 (29%)
At 60 years of age	16 (51.6%)
At 70 years of age	6 (19.4%)
Do you know the difference between Pap smear and high vaginal swap (n=507)	
Yes	51 (10.1%)
No	456 (89.9%)

Table 4 shows the attitude of all respondents towards Pap smear as a screening test for cervical cancer. A total of 297 women (58.6%) never advised their families/relatives/friends to do Pap smear whereas 429 women (84.6%) never requested their physician to do Pap smear. At the end of the survey, almost all women (95.3%) expressed an interest in knowing more information about the Pap smear screening test.

**Table 4:** Attitudes of all respondents towards Pap smear as a screening test for cervical cancer

Have you ever advised your family/relatives/friends to do Pap smear	
Sometimes	130 (25.6%)
Always	80 (15.8%)
Never	297 (58.6%)
Do you ask your physician to do Pap smear	
Sometimes	61 (12%)
Always	17 (3.4%)
Never	429 (84.6%)
Are you interested in knowing more information about Pap smear	
Yes	483 (95.3%)
No	24 (4.7%)

## Discussion

Cervical cancer is a preventable gynecological disease, and an essential facet of its prevention is the advantageous early identification of premalignant lesions by Pap smear [16]. It is also highly curable when diagnosed early [15]. Despite being preventable and curable, most women in Saudi Arabia present to clinical attention with advanced stages that necessitate aggressive multidisciplinary management strategies, including surgery, radiotherapy and chemotherapy [17]. The primary reason for such advanced disease presentations can be largely attributed to lack of intensive screening program for cervical cancer [13] and absence of formal nationwide campaigns to vaccinate females against HPV [14].

Despite the vast majority of respondents were university educated women (45.6%), it is worrisome that great proportions of them were not aware of the Pap smear screening test (46.2%), its importance (61.6 %) and had not done it before (75.2%). These figures raise alarming concerns about the awareness efforts played by the Ministry of Health, individual healthcare facilities, practicing physicians and public media. The finding of highly educated women among this study respondents is generally not anticipated in a normally distributed population from a developing third-world country, and the awareness level could be actually much worse than what was presented in the study. A directly proportional correlation between the low level of education and low level of awareness of Pap smear has been documented previously [18].

HPV is the most frequent sexually transmitted virus and its infection is responsible for nearly 99% of all the cases of cervical cancer [19]. The vast majority of respondents were young (88%) and married (94.1%) women, which imply a population who is sexually active with potentially higher risk of HPV infection and development of cervical cancer. Hence, in this predominant population of sexually active women, Pap smear as a screening test for cervical cancer is warranted and greatly advised to protect themselves and their partners [12].

Our study demonstrated that the current practice of Pap smear screening is beneficially ‘opportunistic’. That is, the healthcare staff gains benefit from women attending healthcare facilities for various obstetrical/gynecologic reasons and offer them Pap smear as a screening test for cervical cancer. The vast majority of women who had Pap smear screening tests (24.9%) were those who attended healthcare facilities for various obstetric/gynecologic reasons other than specific reasons of screening for cervical cancer. Therefore, women who do not physically attend healthcare facilities, irrespective of the reasons, are unlikely to receive Pap smear. This issue is further complicated by the fact that both women (84.2%) and physicians (75.5%) never asked the other party about Pap smear. As a result, a great proportion of women never did Pap smear previously (75.2%). This issue highlights a two-sided (patients and physicians) lack of awareness about Pap smear as a valuable screening test for cervical cancer. It is extremely crucial that women should be well-knowledgeable, self-motivated and intrinsically-driven to attend healthcare facilities for primary reasons to undergo Pap smear. An earlier study in Saudi Arabia revealed that nearly 91% of gynecological and non-gynecological physicians were aware of Pap smear as a screening method for cervical cancer [20]. However, only 35% of them sent/advised some patients for Pap smear testing. It must be noted that physicians play vital roles in contributing success to this mission by disseminating the practice of Pap smear among their eligible patients. It can be inferred that there is an urgent need for proper wide-spread awareness campaigns that highlight the importance of such Pap smear screening to both women and physicians.

Also, our study demonstrated poor knowledge and misinformation about Pap smear by the survey respondents. Our findings mirrored others findings reported elsewhere in Saudi Arabia among university students specifically [14-15] and public women generally [13]. Despite the unsatisfactory knowledge, the survey respondents expressed positive attitudes towards knowing more about the Pap smear test. Various means can be effectively utilized to achieve proper education about Pap smear, such as curricular education in universities, awareness campaigns, pamphlets, booklets, internet, television, radio and healthcare staff. Medical students, in particular, are the future healthcare workforces and can play instrumental roles in increasing awareness of Pap smear among the general population. Correct knowledge about Pap smear test (with respect to when to start, when to stop and frequency of testing) is essential to ensure maximal benefit and adherence to the preventive measures implicated in the Pap smear screening test.

## Conclusions

The awareness and knowledge of Pap smear as a screening test for cervical cancer among Saudi population in Riyadh is unsatisfactory. There is an urgent necessity to educate and foster awareness concerning cervical cancer, its screening and prevention. The education should start as early as possible in primary healthcare centers and secondary schools/universities. In the midst of the economic situation and as the healthcare system cannot afford the high costs implicated in advanced cervical cancer management, a formal intensive screening and HPV vaccination programs for cervical cancer should be launched and supported. All available resources (healthcare facilities, clinical staff, media, etc) should be utilized effectively. These preventive programs should be accessible to all populations (irrespective of socioeconomic status and geographical site) and provide all possible support to women (financially, emotionally, psychologically, etc). Most importantly, interpretation of Pap smear results and their implications should be well educated to women. Also, we plead healthcare policy makers to establish the Saudi Arabian Cervical Screening Registry with call and recall system to ensure including most of the Saudi women. This registry will provide important epidemiological and clinicopathological database that will be extremely helpful in devising strategies to increase awareness. Until these preventive programs (Pap smear and HPV vaccination) are established firmly in place, all healthcare professionals (primary and obstetrician/gynecologist physicians) should be continuously advised to practice ‘opportunistic’ Pap smear screening for all eligible women who attend their healthcare facilities. Regular follow-up is equally important to obviate cervical cancer-related morbidity and mortality.
